# Determination of Antioxidant, Antidiabetic, Anticholinergic, Antiglaucoma Properties and Comprehensive Phytochemical Content by LC‐MS/MS of Bingöl Honeybee Pollen

**DOI:** 10.1002/fsn3.4531

**Published:** 2025-03-03

**Authors:** Ebubekir İzol, Münire Turhan, Mustafa Abdullah Yılmaz, Cüneyt Çağlayan, İlhami Gülçin

**Affiliations:** ^1^ Bee and Natural Products R&D and P&D Application and Research Center Bingöl University Bingöl Türkiye; ^2^ Faculty of Agriculture, Vocational School of Food, Agriculture and Livestock Bingöl University Bingöl Türkiye; ^3^ Department of Pharmaceutical Chemistry, Faculty of Pharmacy Dicle University Diyarbakır Türkiye; ^4^ Department of Medical Biochemistry, Faculty of Medicine Bilecik Şeyh Edebali University Bilecik Türkiye; ^5^ Department of Chemistry, Faculty of Science Ataturk University Erzurum Türkiye

**Keywords:** anticholinergic, antidiabetic, antiglaucoma, antioxidant, bee pollen, phytochemical content

## Abstract

Honeybee pollen is the most important food and protein source for bees. It is a highly nutritious natural product for humans due to its content. Pollen shows different phytochemical content and variable biological activity according to different geography and vegetation. Therefore, in this study, the comprehensive phytochemical content by LC‐MS/MS, and its antioxidant properties by different assays, with enzyme inhibition potential; antidiabetic, anticholinergic, and antiglaucoma properties, were determined from a pollen sample from Bingöl province, one of the significant beekeeping centers in Türkiye. As a result of LC‐MS/MS, 15 metabolites were determined, and the highest concentration of quinic acid (1.531 mg analyte/g extract) was found. Antioxidant results: total phenolic content 113.14 mg GAE/g, total flavonoid content 64.11 mg QE/g, Fe^3+^ reduction 0.43 μg/mL, CUPRAC 0.511 μg/mL, FRAP 0.976 μg/mL, DPPH IC_50_: 1.06 μg/mL, ABTS IC_50_: 0.933 μg/mL, and DMPD IC_50_: 0.598 μg/mL. In addition, pollen showed antiglaucoma, anticholinergic, and antidiabetic properties by inhibiting carbonic anhydrase isoenzyme II (hCA II), acetylcholinesterase (AChE), and α‐ amylase enzymes, respectively. In this study, it was determined that Bingöl pollen has a comprehensive phytochemical content and antioxidant properties and, for the first time, inhibits hCA II, AChE, and α‐amylase enzymes.

## Graphical abstract

Phytochemical content and comprehensive biological activities of Bingöl bee pollen were determined for the first time in this study. Enzyme inhibition and antioxidant studies are valuable.

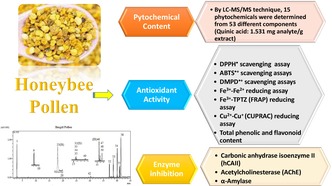

## 1 Introduction

Bee pollen is the substance that bees collect from the male seminal organ in flowers on their feet, pelletize with digestive enzymes, and accumulate in the hive (Taha et al. 2024). Bee pollen is necessary to meet the basic nutritional needs of honey bees, especially protein, lipid, sterol, vitamin, and mineral substances (Kolayli et al. 2024). Bee pollen is a balanced food not only for bees but also for humans. It contains protein (10%–40%), lipids (1%–12%), carbohydrates (13%–55%), fiber (0.3%–20%), mineral substances (0.2%–3.0%), and other organic compounds. It also contains all essential amino acids and fatty acids, B vitamins, carotenoids, and many different polyphenols, as well as important sugars such as fructose, glucose, sucrose, maltose, isomaltose, arabinose, melibiose, trehalose, and starch (Glavnik et al. 2024). Due to its excellent nutritional properties and therapeutic functions, it is one of the most natural food supplements for athletes, children, the elderly and malnourished individuals (El‐Seedi et al. 2022). Bee pollen consumption is also reported to be beneficial to human health as it is effective against immune system strengthening (Münstedt et al. 2015), cancer and allergic diseases (Denisow and Denisow‐Pietrzyk 2016), skin diseases and liver damage (Liang et al. 2020), prostatitis (Denisow and Denisow‐Pietrzyk 2016), diabetes (Lima, Brito, and da Cruz Nizer 2021), immune system damage, and infectious diseases, as well as improving ovarian function and improving neuroinflammation (Saral et al. 2022). In general, in vivo and in vitro scientific studies have shown that bee pollen exhibits good antioxidant, antiviral, anti‐inflammatory, and antitumoral properties (Silveira et al. 2024; Çobanoğlu 2024). However, it has also been shown that all these biological activities vary depending on the specific structure of the pollen. Bee pollen is a natural product with high nutritional and medicinal value. This is due to its protein, mineral, and polyphenolic components (Dos Santos et al. 2024).

Antioxidants are substances that do not form radicals by removing free radicals and oxidative stress (Gülcin 2012). Antioxidants act as pioneers in the treatment of diseases and are used in the treatment of more than a hundred diseases (Gulcin 2020; Karagecili et al. 2023; Karageçili et al. 2023). Due to the damages caused by synthetic antioxidants, intensive studies have been carried out to determine natural antioxidants (Yilmaz et al. 2023). They are also effective in the protection of food because they prevent oxidation (Gülçin et al. 2020). For this reason, it is important to determine the antioxidant properties of Bingöl pollen.

Enzymes such as antioxidants play important roles in the treatment of diseases. hCA II enzyme has been found to be associated with glaucoma disease (Özaslan et al. 2022; Inci et al. 2023), AChE with Alzheimer's disease (Izol et al. 2021; Tokalı et al. 2023) and α‐amylase enzyme with diabetes (Kiziltas et al. 2022), and these enzymes are used in the treatment of these diseases. Therefore, it is very important to determine the phytochemical content of pollen, a natural bee product, and the inhibition potential of these enzymes.

In this study, the phytochemical content by LC‐MS/MS, some metabolic enzyme inhibition (AChE, hCA II, and α‐amylase) and antioxidant activities of pollen obtained from Bingöl, an important beekeeping city in Türkiye, were determined for the first time in such a comprehensive manner. The anti‐Alzheimer's disease, antiglaucoma and antidiabetic properties and antioxidant potential of such Bingöl pollen were determined by different methods. In addition, its phytochemical content was screened with 53 different components and found to contain important polyphenols.

## 2 Materials And Methods

### 2.1 Chemicals

Standard phytochemical substances were purchased from Sigma‐Aldrich (Steinheim, Germany) for use in LC‐MS/MS analysis. Commercial purchases of standards and other chemicals were made from Sigma‐Aldrich Chemie GmbH (Steinheim, Germany) for use in antioxidant and enzyme experiments. The hCA II enzyme was purified by Izol using human erythrocytes and the Sepharose‐4B tyrosine‐sulfanilamide affinity column method (Inci et al. 2023).

### 2.2 Extraction of Pollen

Pollen was obtained from Bingöl province, which has important beekeeping activities in Türkiye. The coordinate of Bingöl province is 39°02′28″ N 40°40′33″D. Pollen collected from the hive was dried in a dark and cool environment. Then, 45 mL of 70% ethanol solvent was added to 15 g of pollen and mixed for 3 days, and extraction was performed by the maceration method. The solvent was removed from the pollen and mixed in the erlene with a rotary evaporator, and the extract was dried. The extraction yield was calculated as 13%. A stock solution was prepared from the dried extract, and this solution was used in the studies. The concentration of the extracts was adjusted as 1 mg/mL.

### 2.3 Determination of Comprehensive Phytochemical Content by LC‐MS/MS

Chromatographic methods have been used to determine the phytochemical content of pollen, and LC‐MS/MS is one of the most efficient methods and gives results even at low concentrations (Aziza et al. 2024). The phytochemical content of the pollen extract was determined by LC‐MS/MS using a validated method (Yilmaz 2020). Fifty‐three phytochemicals were quantitatively analyzed in this assay using a Shimadzu‐Nexera model ultra‐high performance liquid chromatography (UHPLC) in conjunction with a tandem mass spectrometer. The brand of the device is Shimadzu LCMS‐8040. LabSolutions (Shimadzu) was used as software. The mobile phase (B) was acetonitrile and methanol. The solvent flow rate was 0.5 mL/min, and the injection volume was 5 μL. Analytical parameters are included in the reference method. The pollen extract was dissolved in 5 mg/mL methanol in an ultrasonic water bath, filtered with Whatman filter paper, added to the LC‐MS/MS vial, and analyzed. Analyses were performed in negative mode.

### 2.4 Total Phenolic and Flavonoid Contents

Total phenolic and flavonoid contents of pollen extract were determined in accordance with a previous study (Inci et al. 2023). To determine the total phenolic content, the extract (0.5 mL) was mixed with 1.0 mL folin–ciocalteu solution and 0.5 mL 1% Na_2_CO_3_. The absorbance of the mixtures was measured at 725 nm after 2 h at room temperature. Total phenolic content was calculated using milligrams of gallic acid equivalent (GAE) as reference.

Distilled water (2.3 mL), potassium acetate (0.5 mL, 1.0 M), aluminum chloride (1.5 mL, 10%), and ethanol (1.5 mL, 95%) were added to the pollen stock solution (0.5 mL) to determine the total flavonoid content. The combinations were incubated at room temperature for 30 min before absorbance measurement at 415 nm. Quercetin was used as a standard, and total flavonoid levels were reported as milligrams of quercetin equivalent (QE) per gram of pollen extract.

### 2.5 Radical Scavenging Assays

#### 2.5.1 ABTS ^•+^ Scavenging Ability Assays

Pollen extract was found to have the ability to scavenge 2,2′‐azino‐bis (3‐ethylbenzothiazoline‐sulfonic acid) (ABTS) radicals using Gulcin's technique (Gülçin et al. 2020). In short, K_2_S_2_0_8_ (2.5 mM) oxidized an aqueous solution of ABTS (7.0 mM) to yield the radical cation (ABTS^•+^). A phosphate buffer (0.1 M, pH 7.4) was used to dilute the ABTS^•+^ solution before use, and the absorbance value of the control was calibrated to 0.750 ± 0.025 at 734 nm. Then, 3 mL of pollen stock solution was mixed with 1 mL of ABTS^•+^ solution at various concentrations (20–60 μg/mL). At 734 nm, the absorbance of ABTS^•+^ was measured following a 30‐min incubation period (Re et al. 1999).

#### 2.5.2 DPPH ^•^ Scavenging Ability Assays

Using the Blois method, the 1,1‐diphenyl‐2‐picrylhydrazyl (DPPH∙) scavenging effect of pollen was determined (Blois 1958). To do this, pollen stock solutions were mixed with 1 mL of blue‐colored DPPH∙ solution (0.1 mM) produced in ethanol at varying concentrations (20–60 μg/mL). After 30 min of room temperature incubation, absorbance readings at 517 nm were measured.

#### 2.5.3 DMPD ^•+^ Scavenging Ability Assays

With a few minor adjustments, the N,N‐dimethyl‐phenylenediamine (DMPD) radical scavenging capacity of pollen was assessed using the methodology of Fogliano et al. (1999) as described previously (Gülçin 2008). Antioxidant substances can transfer a hydrogen atom to DMPD^•+^ in the presence of Fe^3+^, which causes the solution to become less colored, as indicated by a drop in absorbance at 505 nm. The DMPD (100 mM) was created by dissolving 209 mg of DMPD in 10 mL of deionized water. Then, 1 mL of this solution was added to 100 mL of 0.1 M acetate buffer (pH 5.25), and 0.2 mL of a 0.05 M ferric chloride (FeCl_3_) solution was added to obtain the colored radical cation (DMPD^•+^). This solution is made fresh every day and has a consistent absorbance for up to 12 h at room temperature. Test tubes were filled with several amounts of pollen (20–60 μg/mL) or conventional antioxidants, and the total volume was brought down to 0.5 mL using distilled water. The absorbance was measured at 505 nm 10 min later. After adding 1 mL of DMPD^•+^ solution straight to the reaction mixture, the absorbance at 505 nm was calculated. As a blank sample, the buffer solution was employed. Calculations were made with the formula given in the previous study (Gülçin et al. 2010).

#### 2.5.4 Determination of IC_50_ Values

The half maximal scavenging concentrations values (IC_50_) were determined using plots of activity (%) versus of pollen. For this purpose, the required graphs were drawn corresponding to the amount of pollen against radicals (DPPH, ABTS^•+^, or DMPD^•+^) scavenging activity at least five different concentrations that would eliminate more than 50% of the existing radicals used in our study. Then, pollen concentrations causing 50% inhibition were calculated from the equations obtained from these graphs (Gulçin and Alwasel 2022, 2023).

### 2.6 Reducing Ability Assays

#### 2.6.1 Cu^2+^ Reducing ‐CUPRAC Assay

By modifying the Apak method, the Cu^2+^ reducing capability of pollen extract was ascertained. (Apak et al. 2008). First, standard antioxidant solutions were added to the tubes after the pollen stock solution was made at various concentrations (20–60 μL). Next, 125 μL of CuCl_2_, 125 μL of neocuproine solutions, and 125 μL of CH_3_COONa (pH:6.5) buffer solution were transferred, in that order. Distilled water was poured into the tubes until a total volume of 1000 μL was reached. Lastly, absorbance values at 450 nm were measured following a half‐hour of darkness (Ak and Gülçin 2008).

#### 2.6.2 Fe^3+^‐Fe^2+^ Reducing

Using a modification of the Oyaizu method, the Fe^3+^‐Fe^2+^ reducing capability of pollen extract was determined (Oyaizu 1986). Pollen extract was used to create standard antioxidant solutions in various quantities (20–60 μL) and a 1 mg/mL stock solution for this purpose. One milliliter of phosphate buffer and 750 μL of distilled water were added to each prepared test tube. Each tube was then filled with 1 mL of [K_3_Fe(CN)_6_] (1%) and left in the dark (40°C) for 30 min. Following the incubation period, 250 mL of FeCl_3_ (0.1%) and 1 mL of TCA (10%) were added to the tubes and mixed. At 700 nm, absorbance values were finally measured (Gülçin 2007).

#### 2.6.3 Fe^3+^‐TPTZ Reducing ‐FRAP Assay

Ultimately, test tubes were filled with pollen stock and standard solutions that had been generated using the Fe^3+^‐TPTZ complex reducing ability method at various concentrations (20–60 μL) (Inci et al. 2023). Subsequently, the contents of these tubes were increased to 500 μL using buffer solution, and 2250 μL solutions of FeCl_3_ and FRAP were added to every tube. After combining 5 mL of tube material with a vortex and letting them sit in the dark for 30 min, the absorbance values at 593 nm were determined (Karagecili et al. 2023; Karageçili et al. 2023).

### 2.7 Enzyme Inhibition Assay

#### 2.7.1 hCA II Enzyme Inhibition

Using sepharose‐4B‐L‐Tyrosine‐sulfanilamide affinity chromatography, the hCA II isoenzyme was isolated from human erythrocyte cells for this experiment. We have previously published the methods (Karagecili et al. 2023; Karageçili et al. 2023). Acetazolamide, a common CA inhibitor, and its stock solution were evaluated at 25°C, 3 min, and 348 nm (Verpoorte, Mehta, and Edsall 1967). Using an activity (%)‐compound plot, the extract CA inhibition potential was determined. The IC_50_ value was calculated based on the activity (%) versus the compound plot.

#### 2.7.2 AChE Enzyme Inhibition

Sigma‐Aldrich Chemie GmbH served as the investigation's commercial source of AChE. The Ellman et al. (1961) approach was used to conduct studies on the inhibition of these enzymes involved in the cholinergic breakdown processes and acetylcholine. Acetylthiocholine iodide (AChI) and 5,5′‐dithiobis (2‐nitrobenzoic acid) (DTNB) were the substrates that were used. Spectrophotometric measurements were made at 412 nm after the reaction components were prepared at different concentrations in the control and sample tubes. Inhibition types and IC_50_ values were determined using the data that was gathered (Izol et al. 2021).

#### 2.7.3 α‐Amylase Enzyme Inhibition

The α‐amylase activity was determined by the method of Xiao, Storms, and Tsang (2006). According to previous research (Taslimi and Gulçin 2017) to prepare a starch solution for this paper, 6 g starch was dissolved in 240 mL 0.4 M NaOH and boiled at 70°C for 25 min. Finally, IC_50_ was determined by determining absorbance values at 580 nm.

### 2.8 Statistical Analysis

Every experiment is run three times for each sample. The data, which are presented as the mean ± SD (*n* = 3), were analyzed using one‐way ANOVA and Tukey's post hoc test; *p* < 0.05 was considered statistically significant.

## 3 Results and Discussion

### 3.1 Phytochemical Content Results by LC‐MS/MS

The results of the quantitative content of 53 different phytochemical components of the pollen sample by LC‐MS/MS are given in Table 1. Since method validation data and analytical parameters are given in the reference method (Yilmaz 2020), only retention time, molecular ions (m/z ratio) of standard analytes, fragment ions, coefficient of determination, limit of detection and limit of quantification, and concentrations of phytochemicals present in pollen are given in this table.

**TABLE 1:** Results of phytochemical components by LC‐MS/MS of pollen sample (mg analyte/g extract).

No	Analyte	Pollen	R.T.	M.I. (m/z)	F.I. (m/z)	*r* ^2^	LOD/LOQ (μg/L)
1	Quinic acid	1.531	3.0	190.8	93.0	0.996	25.7/33.3
2	Fumaric aid	—	3.9	115.2	40.9	0.995	135.7/167.9
3	Aconitic acid	—	4.0	172.8	129.0	0.971	16.4/31.4
4	Gallic acid	—	4.4	168.8	79.0	0.999	13.2/17.0
5	Epigallocatechin	—	6.7	304.8	219.0	0.998	237.5/265.9
6	Protocatechuic acid	—	6.8	152.8	108.0	0.957	21.9/38.6
7	Catechin	—	7.4	288.8	203.1	0.999	55.0/78.0
8	Gentisic acid	—	8.3	152.8	109.0	0.997	18.5/28.2
9	Chlorogenic acid	0.028	8.4	353.0	85.0	0.995	13.1/17.6
10	Protocatechuic aldehyde	—	8.5	137.2	92.0	0.996	15.4/22.2
11	Tannic acid	—	9.2	182.8	78.0	0.999	15.3/22.7
12	Epigallocatechin gallate	—	9.4	457.0	305.1	0.999	61.0/86.0
13	Cynarine	—	9.8	515.0	191.0	0.999	5.8/9.4
14	4‐OH Benzoic acid	—	10.5	137.2	65.0	0.999	68.4/88.1
15	Epicatechin	—	11.6	289.0	203.0	0.996	139.6/161.6
16	Vanillic acid	—	11.8	166.8	108.0	0.999	141.9/164.9
17	Caffeic acid	—	12.1	179.0	134.0	0.999	7.7/9.5
18	Syringic acid	—	12.6	196.8	166.9	0.998	82.3/104.5
19	Vanillin	—	13.9	153.1	125.0	0.996	24.5/30.4
20	Syringic aldehyde	—	14.6	181.0	151.1	0.999	19.7/28.0
21	Daidzine	—	15.2	417.1	199.0	0.996	7.0/9.5
22	Epicatechin gallate	—	15.5	441.0	289.0	0.997	19.5/28.5
23	Piceid	—	17.2	391.0	135/106.9	0.999	13.8/17.8
24	*p*‐Coumaric acid	—	17.8	163.0	93.0	0.999	25.9/34.9
25	Ferulic acid‐D3‐IS	N.A.	18.8	196.2	152.1	N.A.	N.A.
26	Ferulic acid	—	18.8	192.8	149.0	0.999	11.8/15.6
27	Sinapic acid	—	18.9	222.8	193.0	0.999	65.2/82.3
28	Coumarin	—	20.9	146.9	103.1	0.999	214.2/247.3
29	Salicylic acid	—	21.8	137.2	65.0	0.999	6.0/8.3
30	Cyranoside	—	23.7	447.0	284.0	0.997	12.1/16.0
31	Miquelianin	—	24.1	477.0	150.9	0.999	10.6/14.7
32	Rutin‐D3‐IS	N.A.	25.5	612.2	304.1	N.A.	N.A.
33	Rutin	0.042	25.6	608.9	301.0	0.999	15.7/22.7
34	Isoquercitrin	0.028	25.6	463.0	271.0	0.998	8.7/13.5
35	Hesperidin	0.063	25.8	611.2	449.0	0.999	19.0/26.0
36	*o*‐Coumaric acid	—	26.1	162.8	93.0	0.999	31.8/40.4
37	Genistin	—	26.3	431.0	239.0	0.991	14.9/21.7
38	Rosmarinic acid	—	26.6	359.0	197.0	0.999	16.2/21.2
39	Ellagic acid	—	27.6	301.0	284.0	0.999	56.9/71.0
40	Cosmosiin	0.006	28.2	431.0	269.0	0.998	6.3/9.2
41	Quercitrin	—	29.8	447.0	301.0	0.999	4.8/6.4
42	Astragalin	0.177	30.4	447.0	255.0	0.999	6.6/8.2
43	Nicotiflorin	—	30.6	592.9	255.0/284.0	0.999	11.9/16.7
44	Fisetin	—	30.6	285.0	163.0	0.999	10.1/12.7
45	Daidzein	—	34.0	253.0	223.0	0.999	9.8/11.6
46	Quercetin‐D3‐IS	N.A.	35.6	304.0	275.9	N.A.	N.A.
47	Quercetin	0.008	35.7	301.0	272.9	0.999	15.5/19.0
48	Naringenin	0.081	35.9	270.9	119.0	0.999	2.6/3.9
49	Hesperetin	—	36.7	301.0	136.0/286.0	0.999	7.1/9.1
50	Luteolin	0.049	36.7	284.8	151.0/175.0	0.999	2.6/4.1
51	Genistein	—	36.9	269.0	135.0	0.999	3.7/5.3
52	Kaempferol	0.009	37.9	285.0	239.0	0.999	10.2/15.4
53	Apigenin	0.018	38.2	268.8	151.0/149.0	0.998	1.3/2.0
54	Amentoflavone	0.055	39.7	537.0	417.0	0.992	2.8/5.1
55	Chrysin	0.019	40.5	252.8	145.0/119.0	0.999	1.5/2.8
56	Acacetin	0.128	40.7	283.0	239.0	0.997	1.5/2.5

Abbreviations: ‐, Not detected; D3, deuterium isotope 3; FI (*m/z*), fragment ions; IS, internal standard; *LOD*/*LOQ* (μg/L), Limit of detection/quantification; M.I. (*m/z*), Molecular ions of the standard analytes (m/z ratio); N.A., not applicable; R.T., retention time; *r* ^*2*^, coefficient of determination.

**FIGURE 1:**
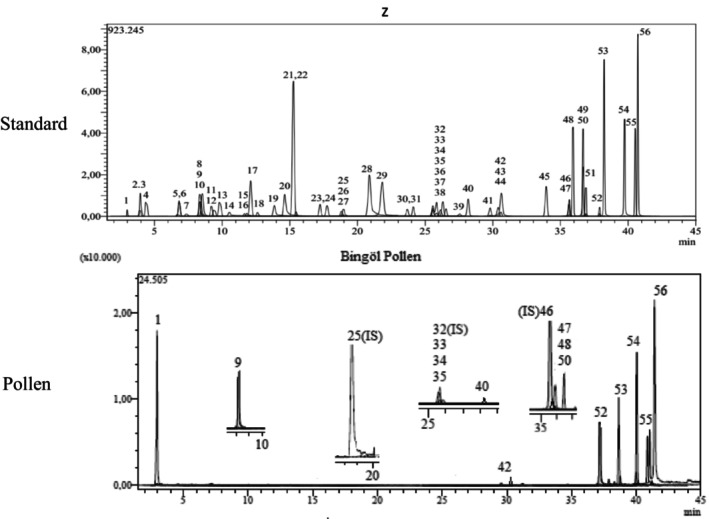
LC‐MS/MS chromatogram of standard phytochemicals and pollen.

LC‐MS/MS chromatograms of standard phytochemicals and pollen are given in Figure 1.

In the pollen sample, 15 components of 53 different phytochemicals were quantitatively determined. Other components could not be detected because they were below the limit of determination. The highest concentration of quinic acid (1.531 mg analyte/g extract) was determined. In general, the phytochemical content of the pollen was not very rich. The chemical forms of the identified compounds are shown in Table 2.

**TABLE 2:** Chemical formula of phytochemicals found in pollen.

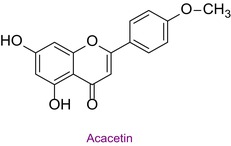	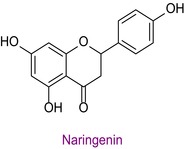	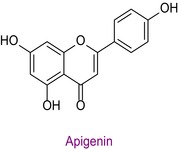
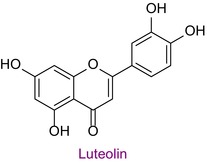	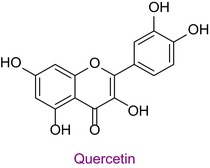	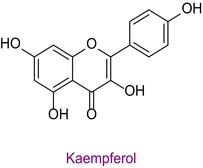
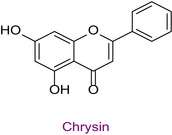	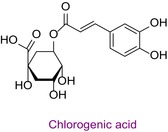	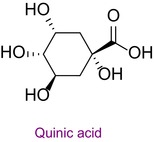
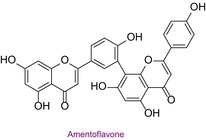	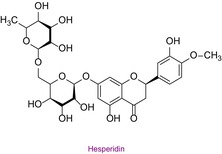	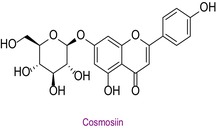
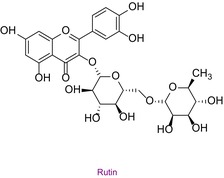	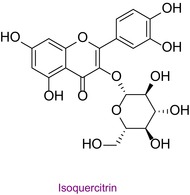	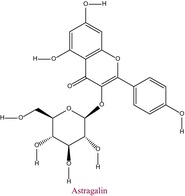

### 3.2 Total Phenolic and Flavonoid Content Result

**FIGURE 2:**
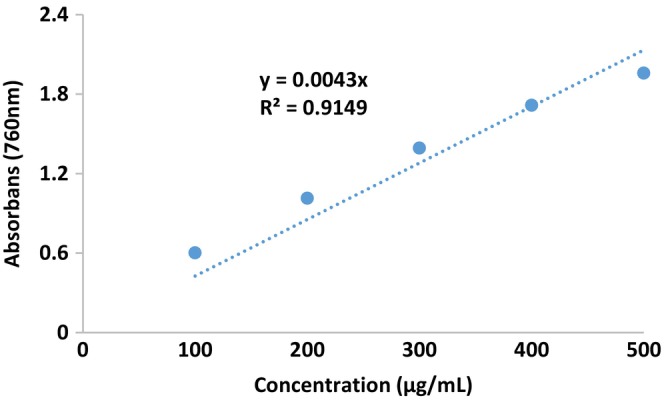
Total phenolic content standard calibration curve graph.

**FIGURE 3:**
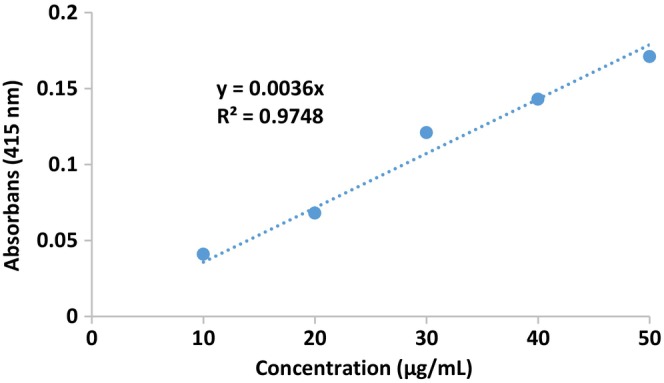
Total flavonoid content standard calibration curve graph.

The results of the total phenolic and total flavonoid content of pollen extract are given in Table 3. The standard calibration graph of the total phenolic amount is given in Figure 2, and the standard calibration graph of the total flavonoid amount is given in Figure 3.

**TABLE 3:** Total phenolic and flavonoid content results of the pollen.

Sample	Pollen
Total phenolics (mg GAE/g)	113.14 ± 3.21
Total flavonoids (mg QE/g)	64.11 ± 1.13

*Note:* The results are given as the mean of three repetitions and ± standard deviation.

### 3.3 Antioxidant Results

The results of the antioxidant reducing assays of pollen extract are given in Table 4. Pollen extract showed lower activity than standard antioxidants in three different antioxidant reducing assays. The results of the antioxidant activity in the Fe^2+^ reducing assay was ranked as α‐tocopherol > trolox > BHA > BHT > pollen. Antioxidant activity results in the Cu^2+^ reducing assay was ranked as BHA > α‐tocopherol > BHT > trolox > pollen. Finally, in the Fe^3+^‐TPTZ reducing assay, the antioxidant activity results were ranked as BHA > trolox > α‐tocopherol > BHT > pollen. It was found that pollen showed antioxidant properties, but this property was below standard antioxidants. Table 5 lists the outcomes of DPPH^•^, ABTS^•+^, and DMPD^•+^ scavenging assays performed on the extract.

**TABLE 4:** Results of antioxidant reduction assays of pollen extract (μg/mL).

Antioxidants and Sample	Fe^3+^ reducing		Cu^2+^ reducing		Fe^3+^‐TPTZ reducing	
	λ_700_	*r* ^2^	λ_450_	*r* ^2^	λ _593_	*r* ^2^
BHA	2.319 ± 0.040	0.9814	2.517 ± 0.106	0.9682	2.142 ± 0.012	0.9933
BHT	1.873 ± 0.151	0.9987	1.488 ± 0.049	0.9823	1.683 ± 0.088	0.9767
Trolox	2.333 ± 0.167	0.9675	1.484 ± 0.068	0.9921	2.041 ± 0.033	0.9799
α‐Tocopherol	2.808 ± 0.272	0.9863	1.509 ± 0.110	0.9897	1.893 ± 0.084	0.9983
Pollen	0.430 ± 0.014	0.9921	0.511 ± 0.011	0.9879	0.976 ± 0.028	0.9895

*Note:* All values are averages of three parallel observations (*n* = 3) and are shown as mean SD (*p* < 0.05 is considered significant). Absorbances were measured at a concentration of 40 μg/mL.

**TABLE 5:** DPPH^•^, ABTS^•+^, and DMPD^•+^ scavenging assays results of pollen at 40 μg/mL.

Antioxidants and Sample	DPPH^•^		ABTS^•+^		DMPD^•+^	
	IC_50_	*r* ^2^	IC_50_	*r* ^2^	IC_50_	*r* ^2^
BHA	0.127 ± 0.008	0.9728	1.079 ± 0.011	0.9877	0.537 ± 0.060	0.9968
BHT	0.492 ± 0.002	0.9877	1.409 ± 0.031	0.9955	1.781 ± 0.062	0.9657
Trolox	0.030 ± 0.002	0.9621	1.472 ± 0.107	0.9721	0.129 ± 0.019	0.9885
α‐Tocopherol	0.341 ± 0.064	0.9975	1.571 ± 0.066	0.9937	0.783 ± 0.015	0.9793
Pollen	1.06 ± 0.089	0.9851	0.933 ± 0.015	0.9894	0.598 ± 0.030	0.9921

*Note:* All values are averages of three parallel observations (*n* = 3) and are shown as mean SD (*p* < 0.05 is considered significant).

According to the DPPH radical scavenging assays, the antioxidant properties of pollen were again found to be lower than standard antioxidants. In DPPH^•^ scavenging assay, the antioxidant activity results were ranked as trolox > BHA > α‐tocopherol > BHT > pollen. In ABTS^**•+**^ scavenging assay, the order was determined as pollen > BHA > BHT > trolox > α‐tocopherol. In ABTS radical scavenging method, the highest antioxidant activity was observed in pollen. In the DMPD^**•+**^ scavenging assay, antioxidant activity was ranked from high to low as trolox > BHA > pollen > BHT > α‐tocopherol. Radical scavenging assays of pollen showed higher antioxidant properties than reducing assays.

### 3.4 Enzyme Inhibition Results

The antiglaucoma property of pollen was revealed by hCA II enzyme inhibition, the anticholinergic property by AChE enzyme inhibition, and the antidiabetic property by α‐amylase enzyme inhibition. Enzyme inhibition results are given in Table 6. Enzyme inhibition results showed that pollen strongly inhibited the enzymes investigated. In three enzyme assays, pollen inhibited more enzymes than the standards. Thus, it was determined that pollen showed strong antidiabetic, antiglaucoma, and anticholinergic properties.

**TABLE 6:** Inhibition values of pollen sample against hCA II isoenzyme, AChE, and α‐amylase enzyme (IC_50_: μg/mL).

Sample and Standard	hCA II		AChE		α‐ Amylase	
	IC_50_	r^2^	IC_50_	r^2^	IC_50_	r^2^
Pollen	0.34	0.9886	0.01	0.9972	0.35	0.9820
Acarbose^a^	—	—	—	—	4.91	0.9763
Tacrine^b^	—	—	4.17^d^	0.9887	3.05^d^	0.9653
Acetazolamide^c^	2.21^d^	0.9712	—	—	—	—

^a^ Acarbose was used as a standard inhibitor for the α‐glycosidase enzyme.

^b^ Tacrin was used as a standard inhibitor for AChE enzyme.

^c^ Acetazolamide was employed as a standard inhibitor hCA II isoenzyme.

^d^ These results is nM.

Phytochemicals are shown to be the main responsible for biological activities. Among these, especially phenolic compounds, are the leading secondary metabolites. Since bee pollen is of plant origin, its phytochemical content is very important in its biological activities. In the study in which the chemical content and antioxidant properties of bee pollen obtained from the Bayburt province of Türkiye were determined, 23 phenolic compounds were screened by LC–MS/MS, and rutin was detected at the highest concentration. In addition, the total phenolic content of pollen was found to be 173.52 mg GA/g, the flavonoid content was 79.21 mg QE/g, and the CUPRAC antioxidant result was 85.59 mg trolox/g (Gercek, Celik, and Bayram 2021). The total phenolic and flavonoid content of Bayburt pollen was higher than that of Bingöl pollen. Polyphenolics investigated as common in Bayburt and Bingöl pollen are gallic acid, protocatechuic acid, caffeic acid, syringic acid, chlorogenic acid, catechin, sinapic acid, p‐coumaric acid, naringenin, ferulic acid, luteolin, quercetin, and kaempferol. Chlorogenic acid, sinapic acid, and naringenin were not detected in Bayburt pollen. Although gallic acid, protocatechuic acid, caffeic acid, syringic acid, salicylic acid, catechin, *p*‐coumaric acid, and ferulic acid were not detected in Bingöl pollen, these compounds were detected in Bayburt pollen. These two studies once again showed the different effects of different geographies and flora on the chemical content and biological activities of bee products.

Total phenolic content was found to be different in different studies. It was found in the range of 44.07–124.2 mg GAE/g in Anzer pollen in Türkiye (Kalaycıoğlu et al. 2017), 41.5–213.2 mg GAE/g in Brazilian pollen (Freire et al. 2012), and 13.53–24.75 mg GAE/g in Italian pollen (Domenici et al. 2015). Total flavonoid content was also found to be different in various geographies. In a study conducted in Türkiye, the total phenolic content, antioxidant properties, and 43 phenolic components of honeybee pollen were investigated. Total phenolic content was found to be between 4.5 and 14.4 mg GAE/g. Antioxidant properties ranged between 94.9 and 233.5 μmol T/g in the ABTS assay and between 25.86 and 70.81 μmol T/g in the DPPH assay. Among the phenolic compounds, rutin, luteolin, kaempferol, genistin, and isorhamnetin were found the most. 29 of 43 phenolic compounds were detected (Çobanoğlu 2024). Chlorogenic acid, rutin, quercetin, naringenin, luteolin, kaempferol, apigenin, and chrysin were found in common with Bingöl pollen. In different studies, honeybee pollen contained luteolin (Karkar, Şahin, and Güneş 2021; Oroian, Dranca, and Ursachi 2022), apigenin (Laaroussi et al. 2020), naringenin, ferulic acid, caffeic acid, vanillic acid (Mohdaly et al. 2015), p‐coumaric acid (Sun et al. 2017), rosmarinic acid (Kahraman et al. 2022), and gallic acid (Adaškevičiūtė et al. 2022) were determined. The total phenolic content of Bingöl pollen was found to be much higher than the pollen in the study. In both studies, it was determined that pollen showed antioxidant properties.

In a different study, the total phenolic content of pollen was found to be 22.98 mg GAE/g, and the total flavonoid content was 33.09 mg RE/g. In addition, 32 different metabolites were detected in total. Among these, quercitrin, kaempferol and naringenin are flavonoid metabolites. The antioxidant properties of pollen were determined by ABTS, DPPH, and FRAP assays. The DPPH result IC_50_ value was 1.17 mg/mL, the ABTS result IC_50_ value was 0.71 mg/mL, and the FRAP result was 113.5 μmol TE/g. In addition, it was determined that the secondary metabolite and antioxidant properties of fermented pollen were higher than those of normal pollen in this study. (Zhang et al. 2023). Since the pollen contents and biological activities of different geographies are different, it is necessary to study the unstudied places and bring the results to the scientific world.

In this study, the antioxidant properties of a pollen sample from Bingöl were determined by eight different assays. In the literature, there is almost no such comprehensive determination of the antioxidant properties of pollen. In addition, since the hCA II, AChE, and α‐amylase enzyme inhibition properties of Bingöl pollen were not reported in the literature, the antiglaucoma, antidiabetic, and anticholinergic potential of this pollen was brought to the literature for the first time. The determination of the phytochemical content of pollen by LC–MS/MS, one of the state‐of‐the‐art techniques, and analysis by a validated method including 53 different components make this study significant. 15 different significant bioactive phytochemicals were identified. Some of them are quinic acid, rutin, hesperidin, quercetin, acacetin, chrysin, apigenin, amentoflavone, and luteolin. Thus, the comprehensive chemical content and antioxidant, antiglaucoma, antidiabetic, and anticholinergic properties of Bingöl pollen were determined for the first time.

## 4 Conclusions

Pollen is a significant natural food source used by humans in their diet. Its high nutrient content has made it a reason for preference. For this reason, the chemical and phytochemical content, antioxidant properties, and enzyme inhibition potentials of pollen are being investigated. In addition, the chemical content, and biological activities of pollen from different geography and flora are different. For this reason, pollen from different geographies is the subject of research. In this study, it was determined that the phytochemical content of honeybee pollen from Bingöl province was moderate, and its antioxidant properties were generally lower than standard antioxidants. In addition, it was determined that it inhibits hCA II, AChE, and α‐amylase enzymes, which are important metabolic enzymes, and therefore shows antiglaucoma, anticholinergic, and antidiabetic properties. Thus, it is predicted that glaucoma, Alzheimer's, and diabetes patients will benefit from consuming Bingöl bee pollen if they do not have any allergies.

## Author Contributions

**Ebubekir İzol:** formal analysis (equal), investigation (equal), methodology (equal), resources (equal), writing – original draft (equal), writing – review and editing (equal). **Münire Turhan:** formal analysis (equal), investigation (equal), methodology (equal). **Mustafa Abdullah Yılmaz:** methodology (equal), software (equal), validation (equal). **Cüneyt Çağlayan:** investigation (equal), methodology (equal), writing – original draft (equal). **İlhami Gülçin:** methodology (equal), writing – original draft (equal), writing – review and editing (equal).

## Conflicts of Interest

The authors declare no conflicts of interest.

## Data Availability

The document contains the necessary data.
